# Tobacco Product Use Among Adults — United States, 2019

**DOI:** 10.15585/mmwr.mm6946a4

**Published:** 2020-11-20

**Authors:** Monica E. Cornelius, Teresa W. Wang, Ahmed Jamal, Caitlin G. Loretan, Linda J. Neff

**Affiliations:** 1 Office on Smoking and Health, National Center for Chronic Disease Prevention and Health Promotion, CDC.

Cigarette smoking remains the leading cause of preventable disease and death in the United States (*1*). The prevalence of current cigarette smoking among U.S. adults has declined over the past several decades, with a prevalence of 13.7% in 2018 (*2*). However, a variety of combustible, noncombustible, and electronic tobacco products are available in the United States (*1*,*3*). To assess recent national estimates of tobacco product use among U.S. adults aged ≥18 years, CDC analyzed data from the 2019 National Health Interview Survey (NHIS). In 2019, an estimated 50.6 million U.S. adults (20.8%) reported currently using any tobacco product, including cigarettes (14.0%), e-cigarettes (4.5%), cigars (3.6%), smokeless tobacco (2.4%), and pipes* (1.0%).^†^ Most current tobacco product users (80.5%) reported using combustible products (cigarettes, cigars, or pipes), and 18.6% reported using two or more tobacco products.^§^ The prevalence of any current tobacco product use was higher among males; adults aged ≤65 years; non-Hispanic American Indian/Alaska Native (AI/AN) adults; those whose highest level of educational attainment was a General Educational Development (GED) certificate; those with an annual household income <$35,000; lesbian, gay, or bisexual (LGB) adults; uninsured adults and those with Medicaid; those with a disability; or those with mild, moderate, or severe generalized anxiety disorder. E-cigarette use was highest among adults aged 18–24 years (9.3%), with over half (56.0%) of these young adults reporting that they had never smoked cigarettes. Implementing comprehensive, evidence-based, population level interventions (e.g., tobacco price increases, comprehensive smoke-free policies, high-impact antitobacco media campaigns, and barrier-free cessation coverage), in coordination with regulation of the manufacturing, marketing, and sale of all tobacco products, can reduce tobacco-related disease and death in the United States (*1*,*4*). As part of a comprehensive approach, targeted interventions are also warranted to reach subpopulations with the highest prevalence of use, which might vary by tobacco product type.

NHIS is an annual, nationally representative, household survey of the noninstitutionalized U.S. civilian population.^¶^ The 2019 NHIS Sample Adult component included 31,997 adults aged ≥18 years; the response rate was 59.1% (*5*). Data were weighted to account for complex survey design and provide nationally representative estimates. Use of five tobacco product types was assessed: cigarettes, cigars (cigars, cigarillos, or filtered little cigars), pipes (regular pipes, water pipes, or hookahs), e-cigarettes, and smokeless tobacco (chewing tobacco, snuff, dip, snus, or dissolvable tobacco). Current cigarette smokers reported having smoked ≥100 cigarettes during their lifetime and reported that they smoked “every day” or “some days” at the time of survey. Current users of all other tobacco products reported using these products “every day” or “some days” at the time of survey. Prevalence estimates for current use of each tobacco product type, any tobacco product, any combustible tobacco product, and two or more tobacco products were calculated. Estimates were calculated overall and by sex, age, race/ethnicity, U.S. Census region,** education (adults aged ≥25 years), marital status, annual household income,^††^ sexual orientation,^§§^ health insurance coverage,^¶¶^ disability status,*** and indication of generalized anxiety disorder (GAD-7).^†††^ The distribution of age groups was assessed among current users of each tobacco product, any tobacco product, combustible products, and two or more tobacco products. Among e-cigarette users, the percentage of current,^§§§^ former,^¶¶¶^ and never**** cigarette smokers was assessed by age group. SAS-callable SUDAAN software (version 11.0.3; RTI International) was used to conduct all analyses.

Among U.S. adults in 2019, 20.8% (estimated 50.6 million) currently used any tobacco product, 16.7% (40.8 million) used any combustible tobacco product, and 3.9% (9.4 million) used two or more tobacco products (Table). Cigarettes were the most commonly used tobacco product (14.0%; 34.1 million). Prevalence of use of other tobacco products was as follows: e-cigarettes (4.5%; 10.9 million); cigars (3.6%; 8.7 million); smokeless tobacco (2.4%; 5.9 million); and pipes (1.0%; 2.4 million). Combustible tobacco products were used by 80.5% of current tobacco product users. Use of two or more tobacco products was reported by 18.6% of current tobacco product users.

**TABLE:** Percentage of adults aged ≥18 years who reported tobacco product use “every day” or “some days,” by tobacco product and selected characteristics — National Health Interview Survey, United States, 2019

Characteristic	% (95% CI)							
	Any tobacco product*	Any combustible Product^†^	Cigarettes^§^	Cigars/Cigarillos/Filtered little cigars^¶^	Regular pipe/Water pipe/Hookah**	E-cigarettes^††^	Smokeless tobacco^§§^	≥2 Tobacco products^¶¶^
**Overall**	**20.8 (20.2–21.4)**	**16.7 (16.1–17.3)**	**14.0 (13.5–14.5)**	**3.6 (3.3–3.9)**	**1.0 (0.9–1.1)**	**4.5 (4.2–4.8)**	**2.4 (2.2–2.6)**	**3.9 (3.6–4.2)**
**Sex**								
Male	26.2 (25.3–27.1)	20.1 (19.3–20.9)	15.3 (14.5–16.1)	6.3 (5.8–6.8)	1.5 (1.3–1.7)	5.5 (5.0–6.0)	4.7 (4.2–5.2)	5.7 (5.2–6.2)
Female	15.7 (14.9–16.5)	13.6 (12.9–14.3)	12.7 (12.0–13.4)	1.1 (0.9–1.3)	0.5 (0.4–0.6)	3.5 (3.1–3.9)	0.3 (0.2–0.4)	2.2 (1.9–2.5)
**Age group (yrs)**								
18–24	18.2 (16.2–20.2)	11.2 (9.7–12.7)	8.0 (6.7–9.3)	3.8 (2.8–4.8)	1.7 (1.1–2.3)	9.3 (7.9–10.7)	2.2 (1.4–3.0)	5.2 (4.1–6.3)
25–44	25.3 (24.2–26.4)	20.1 (19.1–21.1)	16.7 (15.8–17.6)	4.4 (3.9–4.9)	1.3 (1.0–1.6)	6.4 (5.8–7.0)	3.2 (2.8–3.6)	5.5 (4.9–6.1)
45–64	23.0 (21.9–24.1)	19.5 (18.5–20.5)	17.0 (16.0–18.0)	3.7 (3.3–4.1)	0.6 (0.4–0.8)	3.0 (2.6–3.4)	2.5 (2.1–2.9)	3.4 (3.0–3.8)
≥65	11.4 (10.6–12.2)	9.9 (9.2–10.6)	8.2 (7.5–8.9)	2.0 (1.6–2.4)	0.5 (0.3–0.7)	0.8 (0.6–1.0)	1.2 (0.9–1.5)	1.3 (1.0–1.6)
Race/Ethnicity***								
White, non–Hispanic	23.3 (22.5–24.1)	18.3 (17.6–19.0)	15.5 (14.8–16.2)	3.8 (3.5–4.1)	1.0 (0.8–1.2)	5.1 (4.7–5.5)	3.4 (3.1–3.7)	4.5 (4.1–4.9)
Black, non–Hispanic	20.7 (19.0–22.4)	18.6 (17.0–20.2)	14.9 (13.4–16.4)	4.4 (3.5–5.3)	1.1 (0.7–1.5)	3.4 (2.6–4.2)	0.5 (0.3–0.7)	3.3 (2.5–4.1)
Asian, non–Hispanic	11.0 (9.0–13.0)	8.6 (6.7–10.5)	7.2 (5.4–9.0)	1.2 (0.6–1.8)	––^†††^	2.7 (1.7–3.7)	––	1.4 (0.8–2.0)
American Indian/Alaska Native, non–Hispanic	29.3 (16.4–42.2)	22.3 (10.5–34.1)	20.9 (9.9–31.9)	––	––	––	––	––
Hispanic	13.2 (11.9–14.5)	11.2 (10.0–12.4)	8.8 (7.8–9.8)	3.0 (2.3–3.7)	0.8 (0.5–1.1)	2.8 (2.2–3.4)	0.5 (0.3–0.7)	2.2 (1.7–2.7)
Other, non–Hispanic	28.1 (23.4–32.8)	22.0 (17.7–26.3)	19.7 (15.7–23.7)	3.1 (1.6–4.6)	––	9.3 (6.0–12.6)	––	7.5 (4.7–10.3)
**U.S. Census region** ^§§§^								
Northeast	18.5 (17.1–19.9)	16.0 (14.7–17.3)	12.8 (11.5–14.1)	3.8 (3.1–4.5)	0.8 (0.5–1.1)	3.3 (2.7–3.9)	1.1 (0.7–1.5)	2.9 (2.4–3.4)
Midwest	23.7 (22.2–25.2)	19.1 (17.8–20.4)	16.4 (15.2–17.6)	3.9 (3.2–4.6)	1.0 (0.7–1.3)	4.5 (3.9–5.1)	3.1 (2.5–3.7)	4.1 (3.5–4.7)
South	22.9 (21.8–24.0)	18.2 (17.2–19.2)	15.4 (14.5–16.3)	3.9 (3.4–4.4)	1.0 (0.8–1.2)	4.9 (4.3–5.5)	3.0 (2.6–3.4)	4.5 (4.0–5.0)
West	16.4 (15.3–17.5)	12.6 (11.6–13.6)	10.4 (9.4–11.4)	2.6 (2.2–3.0)	1.0 (0.7–1.3)	4.4 (3.8–5.0)	1.9 (1.4–2.4)	3.4 (2.9–3.9)
**Education (adults aged ≥25 years)**								
0–12 years (no diploma)	26.4 (24.2–28.6)	23.5 (21.4–25.6)	21.6 (19.5–23.7)	3.0 (2.1–3.9)	1.2 (0.6–1.8)	3.0 (2.2–3.8)	2.9 (2.1–3.7)	4.0 (3.1–4.9)
General Educational Development	43.7 (39.1–48.3)	37.1 (32.8–41.4)	35.3 (31.1–39.5)	5.2 (3.2–7.2)	––	7.8 (5.5–10.1)	4.9 (2.6–7.2)	8.9 (6.4–11.4)
High school diploma	26.4 (25.0–27.8)	21.9 (20.6–23.2)	19.6 (18.3–20.9)	3.7 (3.1–4.3)	0.8 (0.6–1.0)	4.3 (3.7–4.9)	3.5 (2.9–4.1)	4.8 (4.1–5.5)
Some college, no diploma	24.8 (23.2–26.4)	20.6 (19.1–22.1)	17.7 (16.3–19.1)	3.7 (2.9–4.5)	0.9 (0.6–1.2)	5.0 (4.2–5.8)	2.0 (1.5–2.5)	3.9 (3.2–4.6)
Associate degree (academic or technical/vocational)	21.2 (19.6–22.8)	16.8 (15.4–18.2)	14.0 (12.7–15.3)	3.8 (3.1–4.5)	0.7 (0.3–1.1)	4.5 (3.7–5.3)	2.8 (2.2–3.4)	4.0 (3.3–4.7)
Undergraduate degree (bachelor’s)	13.1 (12.2–14.0)	10.0 (9.1–10.9)	6.9 (6.2–7.6)	3.4 (2.8–4.0)	0.9 (0.6–1.2)	3.2 (2.7–3.7)	1.5 (1.2–1.8)	2.4 (2.0–2.8)
Graduate degree (master's, professional, or doctoral)	8.7 (7.8–9.6)	7.1 (6.2–8.0)	4.0 (3.3–4.7)	3.2 (2.6–3.8)	0.7 (0.4–1.0)	1.5 (1.1–1.9)	1.0 (0.7–1.3)	1.5 (1.1–1.9)
**Marital status**								
Married/Living with partner	19.2 (18.5–19.9)	15.3 (14.6–16.0)	12.4 (11.8–13.0)	3.5 (3.1–3.9)	0.8 (0.6–1.0)	3.9 (3.5–4.3)	2.5 (2.2–2.8)	3.2 (2.9–3.5)
Divorced/Separated/Widowed	23.5 (22.2–24.8)	20.6 (19.4–21.8)	19.0 (17.9–20.1)	3.0 (2.5–3.5)	0.8 (0.5–1.1)	3.3 (2.8–3.8)	2.1 (1.7–2.5)	4.2 (3.6–4.8)
Single/Never married/Not living with a partner	23.0 (21.6–24.4)	17.8 (16.5–19.1)	14.6 (13.4–15.8)	4.1 (3.5–4.7)	1.7 (1.3–2.1)	6.9 (6.1–7.7)	2.5 (1.9–3.1)	5.3 (4.6–6.0)
**Annual household income ($)** ^¶¶¶^								
<35,000	27.0 (25.7–28.3)	23.2 (22.0–24.4)	21.4 (20.2–22.6)	3.2 (2.8–3.6)	1.2 (0.9–1.5)	5.0 (4.4–5.6)	2.0 (1.6–2.4)	4.8 (4.2–5.4)
35,000–74,999	22.0 (20.9–23.1)	18.1 (17.1–19.1)	15.7 (14.7–16.7)	3.2 (2.7–3.7)	1.1 (0.8–1.4)	4.5 (4.0–5.0)	2.5 (2.1–2.9)	4.3 (3.8–4.8)
75,000–99,999	18.8 (17.3–20.3)	14.5 (13.1–15.9)	11.4 (10.1–12.7)	3.9 (3.1–4.7)	1.1 (0.6–1.6)	4.6 (3.7–5.5)	2.4 (1.8–3.0)	3.5 (2.7–4.3)
≥100,000	15.1 (14.1–16.1)	10.8 (10.0–11.6)	7.1 (6.4–7.8)	4.1 (3.6–4.6)	0.7 (0.5–0.9)	3.8 (3.3–4.3)	2.7 (2.2–3.2)	2.8 (2.4–3.2)
**Sexual orientation**								
Heterosexual/Straight	20.5 (19.9–21.1)	16.5 (15.9–17.1)	13.8 (13.2–14.4)	3.6 (3.3–3.9)	0.9 (0.8–1.0)	4.2 (3.9–4.5)	2.5 (2.3–2.7)	3.8 (3.5–4.1)
Lesbian, gay, or bisexual	29.9 (25.9–33.9)	22.7 (19.2–26.2)	19.2 (16.1–22.3)	4.7 (2.9–6.5)	2.3 (1.1–3.5)	11.5 (8.7–14.3)	––	6.9 (5.0–8.8)
Health insurance coverage****								
Private insurance	18.0 (17.3–18.7)	13.7 (13.1–14.3)	10.7 (10.1–11.3)	3.6 (3.3–3.9)	0.9 (0.7–1.1)	4.3 (3.9–4.7)	2.5 (2.2–2.8)	3.3 (3.0–3.6)
Medicaid	30.0 (27.9–32.1)	26.8 (24.8–28.8)	24.9 (22.9–26.9)	3.3 (2.6–4.0)	1.1 (0.7–1.5)	5.0 (4.0–6.0)	1.8 (1.3–2.3)	5.3 (4.3–6.3)
Medicare only (aged ≥65 yrs)	11.4 (9.9–12.9)	10.1 (8.7–11.5)	8.6 (7.3–9.9)	1.8 (1.2–2.4)	––	1.0 (0.6–1.4)	––	1.2 (0.7–1.7)
Other public insurance	25.6 (23.2–28.0)	20.8 (18.7–22.9)	17.8 (15.9–19.7)	5.4 (3.9–6.9)	1.1 (0.6–1.6)	4.4 (3.2–5.6)	3.4 (2.2–4.6)	5.2 (4.0–6.4)
Uninsured	30.2 (28.0–32.4)	24.9 (22.9–26.9)	22.5 (20.6–24.4)	4.1 (3.1–5.1)	1.3 (0.8–1.8)	7.2 (6.1–8.3)	2.9 (2.1–3.7)	6.5 (5.4–7.6)
**Disability** ^††††^								
Yes	26.9 (24.9–28.9)	23.1 (21.2–25.0)	21.1 (19.3–22.9)	3.7 (2.8–4.6)	1.4 (0.9–1.9)	4.2 (3.3–5.1)	2.8 (2.1–3.5)	5.0 (4.1–5.9)
No	20.1 (19.5–20.7)	16.1 (15.5–16.7)	13.3 (12.8–13.8)	3.6 (3.3–3.9)	0.9 (0.8–1.0)	4.5 (4.2–4.8)	2.4 (2.1–2.7)	3.8 (3.5–4.1)
**Generalized anxiety disorder** ^§§§§^								
None/Minimal	18.4 (17.8–19.0)	14.7 (14.1–15.3)	12.0 (11.5–12.5)	3.4 (3.1–3.7)	0.8 (0.7–0.9)	3.6 (3.3–3.9)	2.4 (2.1–2.7)	3.2 (2.9–3.5)
Mild	30.4 (28.3–32.5)	24.3 (22.3–26.3)	21.5 (19.5–23.5)	4.0 (3.0–5.0)	1.6 (1.1–2.1)	8.9 (7.5–10.3)	2.3 (1.6–3.0)	6.6 (5.4–7.8)
Moderate	34.2 (30.7–37.7)	29.2 (25.9–32.5)	27.0 (23.8–30.2)	3.9 (2.4–5.4)	2.6 (1.2–4.0)	9.6 (7.3–11.9)	2.2 (1.1–3.3)	8.2 (6.1–10.3)
Severe	45.3 (41.1–49.5)	38.7 (34.5–42.9)	34.5 (30.5–38.5)	6.7 (4.5–8.9)	2.1 (1.0–3.2)	10.1 (7.5–12.7)	3.5 (1.9–5.1)	9.4 (6.9–11.9)

**Abbreviation:** CI = confidence interval.

* Any tobacco use was defined as use either “every day” or “some days” of at least one tobacco product. (For cigarettes, users were defined as adults who reported use either “every day” or “some days” and had smoked ≥100 cigarettes during their lifetime).

^†^ Any combustible tobacco use was defined as use either “every day” or “some days” of at least one combustible tobacco product: cigarettes; cigars, cigarillos, filtered little cigars; pipes, water pipes, or hookah. (For cigarettes, users were defined as adults who reported use either “every day” or “some days” and had smoked ≥100 times during their lifetime).

^§^ Current cigarette smokers were defined as adults who reported smoking ≥100 cigarettes during their lifetime and now smoked cigarettes “every day” or “some days.”

^¶^ Current cigar smokers were defined as adults who reported smoking cigars, cigarillos, or little filtered cigars at least once during their lifetime and now smoked at least one of these products “every day” or “some days.”

** Current pipe smokers were defined as adults who reported smoking tobacco in a regular pipe, water pipe, or hookah at least once during their lifetime and now smoked at least one of these products “every day” or “some days.”

^††^ Current electronic cigarette (e-cigarette) users were reported as adults who reported using e-cigarettes at least once during their lifetime and now used e-cigarettes “every day” or “some days.”

^§§^ Current smokeless tobacco product users were defined as adults who reported using chewing tobacco, snuff, dip, snus, or dissolvable tobacco at least once during their lifetime and now used at least one of these products “every day” or “some days.”

^¶¶^ Current multiple tobacco product users were defined as adults who reported use “every day” or “some days” for at least two or more of the following tobacco products: cigarettes (≥100 cigarettes during lifetime); cigars, cigarillos, filtered little cigars; pipes, water pipes, or hookah; e-cigarettes; or smokeless tobacco products.

*** Hispanic adults could be of any race. All other groups were non-Hispanic. The following four non-Hispanic single-race categories were available for sample adults in the 2019 NHIS public use files: 1) White; 2) Black or African American; 3) Asian; and 4) American Indian or Alaska Native (AI/AN). Exclusive from these groups, the “Non-Hispanic, Other” category includes those adults who were categorized as “Non-Hispanic AI/AN and any other group” or “other single and multiple races.” The only multiracial category available was “Non-Hispanic AI/AN and any other group.” ftp://ftp.cdc.gov/pub/Health_Statistics/NCHS/Dataset_Documentation/NHIS/2019/srvydesc-508.pdf.

^†††^ Estimates with a relative standard error >30% or unweighted denominator <50 are suppressed.

^§§§^ *Northeast:* Connecticut, Maine, Massachusetts, New Hampshire, New Jersey, New York, Pennsylvania, Rhode Island, and Vermont; *Midwest:* Illinois, Indiana, Iowa, Kansas, Michigan, Minnesota, Missouri, Nebraska, North Dakota, Ohio, South Dakota, and Wisconsin; *South:* Alabama, Arkansas, Delaware, District of Columbia, Florida, Georgia, Kentucky, Louisiana, Maryland, Mississippi, North Carolina, Oklahoma, South Carolina, Tennessee, Texas, Virginia, and West Virginia; *West:* Alaska, Arizona, California, Colorado, Hawaii, Idaho, Montana, Nevada, New Mexico, Oregon, Utah, Washington, and Wyoming.

^¶¶¶^ Based on the imputed sample adult family income (grouped) variable (n = 31,997). ftp://ftp.cdc.gov/pub/Health_Statistics/NCHS/Dataset_Documentation/NHIS/2019/srvydesc-508.pdf.

**** Private insurance: includes adults who had any comprehensive private insurance plan (including health maintenance organizations and preferred provider organizations). Medicaid: For adults aged <65 years, includes adults who do not have private coverage, but who have Medicaid or other state-sponsored health plans including Children’s Health Insurance Program (CHIP); for adults aged ≥65 years, includes adults aged ≥65 years who do not have any private coverage but have Medicare and Medicaid or other state-sponsored health plans. Medicare only: includes adults aged ≥65 years who only have Medicare coverage. Other public insurance: includes adults who do not have private insurance, Medicaid, or other public coverage, but who have any type of military coverage, coverage from other government programs, or Medicare (adults aged <65 years). Uninsured: includes adults who have not indicated that they are covered at the time of the interview under private health insurance, Medicare, Medicaid, a state-sponsored health plan, other government programs, or military coverage. Insurance coverage is “as of time of survey.”

^††††^ Disability was defined based on self-reported presence of selected limitations including vision, hearing, mobility, remembering or concentrating, self-care, and communication. Respondents had to answer “A lot of difficulty” or “Cannot do at all/unable to do” to one of the following questions: “Do you have difficulty seeing, even when wearing glasses?,” “Do you have difficulty hearing, even when using a hearing aid?,” “Do you have any difficulty walking or climbing steps?,” “Using your usual language, do you have difficulty communicating, for example, understanding or being understood?,” “Do you have difficulty remembering or concentrating?,” “Do you have difficulty with self-care, such as washing all over or dressing?” to be coded as having a disability; those who responded “no difficulty” or “some difficulty” to all six questions were coded to not have a disability. Classifications are based on the 2019 NHIS Washington Group Short Set Composite Disability Indicator recode, as based on the short set of questions recommended by the Washington Group on Disability Statistics (https://www.cdc.gov/nchs/washington_group/index.htm).

^§§§§^ Based on the 7-item Generalized Anxiety Disorder Scale (GAD-7) recode of none/minimal (values 0–4), mild (values 5–9), moderate (values 10–14) and severe (values 15–21). Adults were asked how often they have been bothered by the following symptoms in the past 2 weeks: “Feeling nervous, anxious, or on edge”; “Not being able to stop or control worrying”; “Worrying too much about different things”; “Trouble relaxing”; “Being so restless that it’s hard to sit still”; “Becoming easily annoyed or irritable”; and “Feeling afraid as if something awful might happen.” Response options were “not at all,” “several days,” “more than half the days,” and “nearly every day,” scored as 0 to 3 points, respectively, and then summed into a total score.

The tobacco product with the highest percentage of users aged 18–24 (24.5%) and 25–44 years (49.3%) was e-cigarettes (Figure 1). The tobacco product with the highest percentage of users aged 45–64 (40.2%) and ≥65 years (12.3%) was cigarettes. Among current e-cigarette users, 36.9% were current cigarette smokers, 39.5% were former cigarette smokers, and 23.6% were never cigarette smokers (Figure 2). The percentage of e-cigarette users who were never smokers was highest (56.0%) among the 18–24 age group and decreased with increasing age. The percentage of e-cigarette users who were former smokers was lowest (20.5%) among the 18–24 age group and increased with increasing age. Many adults in all age groups were dual users of e-cigarettes and cigarettes.

**FIGURE 1:**
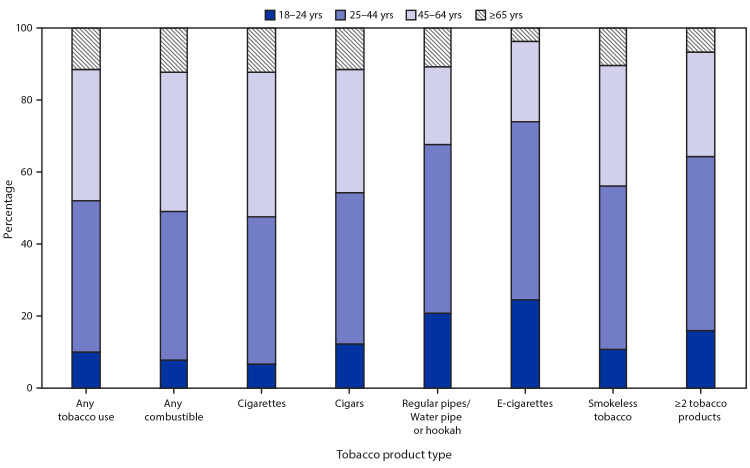
Age distribution of adults aged ≥18 years who reported current tobacco product use* — National Health Interview Survey, United States, 2019 *Any tobacco use was defined as use either “every day” or “some days” of at least one tobacco product among individuals. For cigarettes, users were defined as adults who reported smoking ≥100 cigarettes during their lifetime, and smoked “every day” or “some days” at the time of interview. Any combustible tobacco use was defined as use either “every day” or “some days” of at least one combustible tobacco product: cigarettes; cigars, cigarillos, filtered little cigars; pipes, water pipes, or hookah. Use of two or more tobacco products was defined as adults who reported use “every day” or “some days” of at least two or more of the following tobacco products: cigarettes; cigars, cigarillos, filtered little cigars; pipes, water pipes, or hookah; e-cigarettes; or smokeless tobacco products.

**FIGURE 2:**
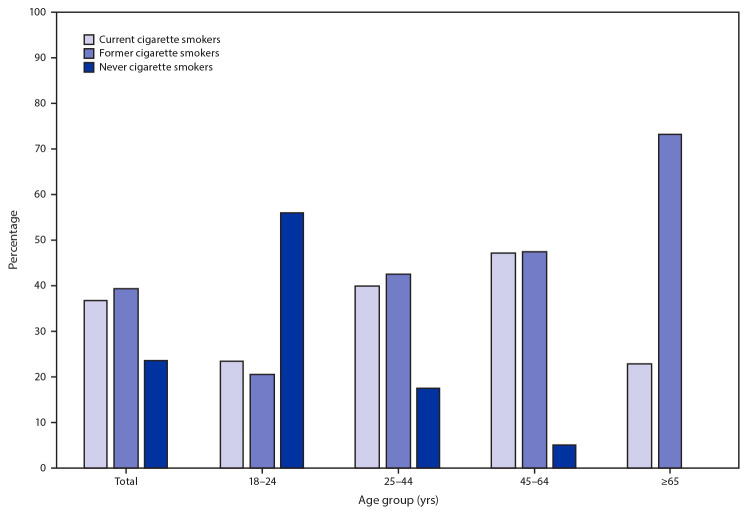
Cigarette smoking status* among current adult e-cigarette users,^†^ by age group^§^ — National Health Interview Survey, United States, 2019 * Adults were asked if they had smoked ≥100 cigarettes in their lifetime and, if yes, whether they currently smoked cigarettes “every day,” “some days,” or “not at all.” Those who smoked “every day” or “some days” were classified as current cigarette smokers. Adults who had not smoked ≥100 cigarettes in their lifetime were classified as never cigarette smokers. Adults who had smoked ≥100 cigarettes in their lifetime but responded to smoking “not at all” at the time of the interview were classified as former cigarette smokers. ^†^ Current e-cigarette users were defined as adults who reported e-cigarette use at least once during their lifetime and use “every day” or “some days” at the time of the interview. **^§^** The prevalence of never cigarette smokers among e-cigarette users aged 65 years and older is not presented because of relative standard error >30% or unweighted denominator <50.

The prevalence of any current tobacco product use was higher among males (26.2%) than among females (15.7%) and among those aged 25–44 years (25.3%), 45–64 years (23.0%), or 18–24 years (18.2%) than among those aged ≥65 years (11.4%) (Table). Current tobacco product use was also higher among non-Hispanic AI/AN adults (29.3%), non-Hispanic adults of other^††††^ races (28.1%), non-Hispanic White adults (23.3%), non-Hispanic Black adults (20.7%), and Hispanic or Latino adults (13.2%) than among non-Hispanic Asian adults (11.0%); and among those living in the Midwest (23.7%) or South (22.9%) than among those in the Northeast (18.5%) or West (16.4%). The prevalence of current tobacco product use was higher among those whose highest educational attainment was a GED (43.7%) than among those with other levels of education; among those who were divorced/separated/widowed (23.5%) or single/never married/not living with a partner (23.0%) than among those married/living with a partner (19.2%); among those who had annual household income of <$35,000 (27.0%) than among those with higher income; and among LGB adults (29.9%) than among those who were heterosexual/straight (20.5%). Prevalence was also higher among adults who were uninsured (30.2%), insured by Medicaid (30.0%), or had some other public insurance (25.6%) than among those with private insurance (18.0%) or Medicare only (11.4%); among those who had a disability (26.9%) compared with those without (20.1%); and among those who had GAD-7 scores indicating mild (30.4%), moderate (34.2%) or severe (45.3%) anxiety than among those indicating no or minimal (18.4%) anxiety.

## Discussion

In 2019, approximately one in five U.S. adults (50.6 million) reported currently using any tobacco product. Cigarettes were the most commonly used tobacco product among adults, and combustible tobacco products (cigarettes, cigars, or pipes) were used by most (80.5%) adult tobacco product users. Most of the death and disease from tobacco use in the United States is primarily caused by cigarettes and other combustible products (*1*); therefore, continued efforts to reduce all forms of combustible tobacco smoking among U.S. adults are warranted. Moreover, approximately one in five current tobacco product users (18.6%) reported using two or more tobacco products, and differences in prevalence of tobacco use were also seen across population groups, with higher prevalence among those with a GED, American Indian/Alaska Natives, uninsured adults and adults with Medicaid, and LGB adults. Each of these groups has experienced social, economic, and environmental stressors that might contribute to higher tobacco use prevalence (*6*). Comprehensive strategies at the national, state, and local levels, including targeted interventions and tailored community engagement, can reduce tobacco-related disease and death and help to mitigate tobacco-related disparities (*1*,*4*,*6*).

U.S. adults also reported using various noncigarette tobacco products, with e-cigarettes being the most commonly used noncigarette tobacco product (4.5%). E-cigarette use was highest among adults aged 18–24 years (9.3%), with over half (56.0%) of these young adults reporting that they had never smoked cigarettes. In addition, the tobacco product with the highest percentage of users aged 18–24 years (24.5%) was e-cigarettes. E-cigarettes contain nicotine, which is highly addictive, can prime the brain for addiction to other drugs, and can harm brain development, which continues until about age 25 years (*3*). Although e-cigarette use was lower among the older age groups, more than 40% of e-cigarette users in the 25–44, 45–64 and ≥65 years age groups reported being former smokers. Although some evidence suggests that the use of e-cigarettes containing nicotine and more frequent use of e-cigarettes are associated with increased smoking cessation, smokers need to completely stop smoking cigarettes and stop using any other tobacco product to achieve meaningful health benefits (*6*,*7*). The U.S. Surgeon General concluded that there is presently inadequate evidence to conclude that e-cigarettes, in general, increase smoking cessation, and further research is needed on the effects that e-cigarettes have on cessation (*7*). Therefore, continued efforts to reduce use of all tobacco products, combustible and noncombustible, are needed.

The findings in this report are subject to at least four limitations. First, the 59.1% response rate might have resulted in nonresponse bias, although sample weighting is designed to account for this. Second, self-reported responses were not validated by biochemical testing for cotinine (a biomarker indicating nicotine exposure); however, there is high correlation between self-reported smoking and smokeless use and cotinine levels (*8*,*9*). Third, because NHIS is limited to the noninstitutionalized U.S. civilian population, these results might not be generalizable to institutionalized populations and persons in the military. Finally, this analysis does not provide comparisons of prevalence estimates with previous surveys because changes in weighting and design methodology for the 2019 NHIS have the potential to affect comparisons of weighted survey estimates over time.^§§§§^

The implementation of comprehensive, evidence-based, population-level interventions in coordination with regulation of tobacco products, can reduce tobacco-related disease, disparities, and death in the United States (*1*,*4*). These evidence-based, population-level strategies include implementation of tobacco price increases, comprehensive smoke-free policies, high-impact antitobacco media campaigns, and barrier-free cessation coverage (*1*). As part of a comprehensive approach, targeted interventions are also warranted to reach subpopulations with the highest prevalence of use, which might vary by tobacco product type.

**Summary**

**What is already known about this topic?**

Cigarette smoking remains the leading cause of preventable disease and death in the United States; however, a variety of new combustible, noncombustible, and electronic tobacco products are available in the United States.

**What is added by this report?**

In 2019, approximately 20.8% of U.S. adults (50.6 million) currently used any tobacco product. Cigarettes were the most commonly used tobacco product among adults, and e-cigarettes were the most commonly used noncigarette tobacco product (4.5%). The highest prevalence of e-cigarette use was among smokers aged 18–24 years (9.3%), with over half (56.0%) of these young adults reporting that they had never smoked cigarettes.

**What are the implications for public health practice?**

The implementation of comprehensive, evidence-based, population-level interventions, combined with targeted strategies, in coordination with regulation of tobacco products, can reduce tobacco-related disease and death in the United States. As part of a comprehensive approach, targeted interventions are also warranted to reach subpopulations with the greatest use, which might vary by tobacco product type.

## Footnotes

* The use of regular pipe, water pipe, or hookah was assessed together using a single question. Interviewers could read the following sentences, if necessary: “A hookah is a type of water pipe. It is sometimes called a narghile pipe. Do not include electronic hookahs or e-hookahs.” “Do not include electronic pipes or e-pipes. Do not include pipes filled with substances other than tobacco.”

† Categories are not mutually exclusive.

§ Current use of two or more tobacco products was defined as “every day” or “some day” use of two or more of the following tobacco products: cigarettes (≥100 cigarettes during lifetime); cigars, cigarillos, or filtered little cigars; pipes, water pipes, or hookahs; electronic cigarettes; or smokeless tobacco products.

¶ https://www.cdc.gov/nchs/nhis/data-questionnaires-documentation.htm.

** *Northeast:* Connecticut, Maine, Massachusetts, New Hampshire, New Jersey, New York, Pennsylvania, Rhode Island, and Vermont. *Midwest:* Illinois, Indiana, Iowa, Kansas, Michigan, Minnesota, Missouri, Nebraska, North Dakota, Ohio, South Dakota, and Wisconsin. *South:* Alabama, Arkansas, Delaware, District of Columbia, Florida, Georgia, Kentucky, Louisiana, Maryland, Mississippi, North Carolina, Oklahoma, South Carolina, Tennessee, Texas, Virginia, and West Virginia. *West:* Alaska, Arizona, California, Colorado, Hawaii, Idaho, Montana, Nevada, New Mexico, Oregon, Utah, Washington, and Wyoming.

†† Based on the imputed sample adult family income (grouped) variable (n = 31,997).

§§ Sexual orientation was determined using the question “Which of the following best represents how you think of yourself?” Response options included “gay,” “straight, that is, not gay,” “bisexual,” “something else,” and “I don’t know the answer” among male respondents, and “lesbian or gay,” “straight, that is, not lesbian or gay,” “bisexual, “something else,” and “I don’t know the answer” among female respondents. Respondents were considered to be lesbian, gay, or bisexual if they responded “gay,” “lesbian or gay,” or “bisexual.”

¶¶ Private coverage: includes adults who had any comprehensive private insurance plan (including health maintenance organizations and preferred provider organizations). Medicaid: for adults aged <65 years, includes those who did not have private coverage, but who had Medicaid or other state-sponsored health plans, including Children’s Health Insurance Program (CHIP). For adults aged ≥65 years, includes adults aged ≥65 years who did not have any private coverage but had Medicare and Medicaid or other state-sponsored health plans; Medicare only: includes adults aged ≥65 years who only had Medicare coverage; Other coverage: includes adults who did not have private insurance, Medicaid, or other public coverage, but who had any type of military coverage, coverage from other government programs, or Medicare. Uninsured: includes adults who did not indicate that they were covered at the time of the interview under private health insurance, Medicare, Medicaid, CHIP, a state-sponsored health plan, other government programs, or military coverage.

*** Disability was defined based on self-reported presence of selected limitations including vision, hearing, mobility, remembering, self-care, communication. Respondents had to answer “A lot of difficulty” or “Cannot do at all/unable to do” to one of the following questions: “Do you have difficulty seeing, even when wearing glasses?,” Do you have difficulty hearing, even when using a hearing aid?,” “Do you have any difficulty walking or climbing steps?,” “Using your usual language, do you have difficulty communicating, for example, understanding or being understood?,” “Do you have difficulty remembering or concentrating?,” “Do you have difficulty with self-care, such as washing all over or dressing?” to be coded as having a disability; those who responded “no difficulty” or “some difficulty” to all six questions were coded as not having a disability. These six questions are based on the short set of questions recommended by the Washington Group on Disability Statistics (https://www.cdc.gov/nchs/washington_group/index.htm).

††† Based on the 7-item Generalized Anxiety Disorder scale (GAD-7) recode of none/minimal (values 0–4), mild (values 5–9), moderate (values 10–14) and severe (values 15–21). Adults were asked how often they have been bothered by the following symptoms in the past 2 weeks: “Feeling nervous, anxious, or on edge”; “Not being able to stop or control worrying”; “Worrying too much about different things”; “Trouble relaxing”; “Being so restless that it’s hard to sit still”; “Becoming easily annoyed or irritable”; and “Feeling afraid as if something awful might happen.” Response options were “not at all,” “several days,” “more than half the days,” and “nearly every day,” scored as 0 to 3 points, respectively, and then summed into a total score.

§§§ Current cigarette smokers were defined as adults who reported smoking ≥100 cigarettes during their lifetime and smoked cigarettes “every day” or “some days” at the time of the interview (only other response options were “not at all, refused, and don’t know”).

¶¶¶ Former cigarette smokers were defined as adults who had smoked ≥100 cigarettes in their lifetime but reported smoking “not at all” at the time of the interview.

**** Never cigarette smokers were defined as adults who had not smoked ≥100 cigarettes in their lifetime.

†††† The following four non-Hispanic single race categories were available for sample adults in the 2019 NHIS public use files: 1) White; 2) Black or African American; 3) Asian; and 4) American Indian or Alaska Native (AI/AN). Exclusive from these groups, the “non-Hispanic, Other” category in this report includes those adults who were categorized as “non-Hispanic AI/AN and any other group” or “other single and multiple races.” The only multiracial category available was “non-Hispanic AI/AN and any other group.” ftp://ftp.cdc.gov/pub/Health_Statistics/NCHS/Dataset_Documentation/NHIS/2019/srvydesc-508.pdf.

§§§§ 2019 NHIS documentation indicates that changes to the nonresponse adjustment approach and the calibration methods for the 2019 NHIS have the potential to affect comparisons of the weighted survey estimates over time. Because of the changes in weighting and design methodology, direct comparisons between estimates for 2019 and earlier years should be made with caution because the effect of these changes has not been fully evaluated at this time. ftp://ftp.cdc.gov/pub/Health_Statistics/NCHS/Dataset_Documentation/NHIS/2019/srvydesc-508.pdf; https://www.cdc.gov/nchs/data/nhis/earlyrelease/EarlyRelease202009-508.pdf.
